# Making Biodegradable Seedling Pots from Textile and Paper Waste—Part A: Factors Affecting Tensile Strength

**DOI:** 10.3390/ijerph18136964

**Published:** 2021-06-29

**Authors:** Jeanger P. Juanga-Labayen, Qiuyan Yuan

**Affiliations:** Environmental Engineering, Civil Engineering Department, University of Manitoba, Winnipeg, MB R3T 5V6, Canada; jeanger.labayen@gmail.com

**Keywords:** textile waste, cotton, paper waste, binder, alkali treatment

## Abstract

This study investigates the efficacy of using discarded textile (cotton and polycotton) and paper waste (newspaper and corrugated cardboard) as substrates to form sheets with optimum tensile strength. The effect of alkali treatment (sodium hydroxide (NaOH) and sodium bicarbonate (NaHCO_3_)), compressive loads (200 N and 500 N), and the use of binding agents (blackstrap molasses, sodium alginate, and cornstarch) were studied to optimize the tensile strength of homogeneous sheets. The alkali treatment using 5% NaOH for 5 h of soaking demonstrated the highest increase in tensile strength of 21% and 19% for cotton and newspaper, respectively. Increasing compressive load from 200 N to 500 N showed the highest increase in tensile strength of 37% and 42% for cotton and newspaper, respectively. Remarkably, among the binders, cornstarch at 20% concentration obtained an increase in tensile strength of 395%, 320%, 310%, and 185% for cotton, polycotton, corrugated cardboard, and newspaper sheets, respectively. The optimum results obtained from this study will be utilized to develop biodegradable seedling pots using discarded textile and paper waste.

## 1. Introduction

Consumerism and economic growth cause an increasing amount of discarded textile and paper waste in municipal solid waste (MSW), which is mostly disposed of in landfills. In 2015, the U.S. generated 16.03 MT (million tons) of textile waste, which is equivalent to 6.11% of the total MSW, where 65.7% (10.5 MT) was landfilled [1]. The primary textile waste in MSW comprises discarded clothing, including non-durable goods such as sheets and towels. Moreover, paper and paperboard waste represents the largest fraction of the total MSW generated (68.05 MT) and recycled (45.32 MT) in the U.S., but it was the 3rd largest fraction of MSW (18.30 MT) disposed of in landfills [1]. Cellulose fibers from paper waste that are recycled 5–6 times become short and weak due to repeated treatment and drying operations [2,3]. Discarded textile and paper waste are considered fiber-rich resources that can be potentially downcycled into valuable products and thereby promote landfill diversion. This study investigates the efficacy of using discarded textile and paper waste as substrates to form sheets with optimum tensile strength by evaluating the effects of alkali treatment, compressive load, and binding agents. 

### 1.1. Alkali Treatment

Natural fibrous waste materials are lignocellulosic, i.e., their primary composition includes cellulose, hemicellulose, and lignin (Table 1). Cellulose has a linear framework with a semicrystalline structure that provides fiber strength, stiffness, and stability; hemicellulose consists of a branched polymer structure that is fully amorphous, and lignin has an aromatic structure that is also amorphous [4,5,6].

Alkaline treatment, or mercerization, of natural fibers is widely used to improve the cellulosic molecular structure that enhances fiber surface adhesion with the binder [4,11]. Alkaline treatment also improves the surface roughness of the fiber by partial removal of hemicellulose, lignin, and other organic substances and distorts the crystalline cellulose structure, thereby increasing reaction sites [12,13]. Several studies have revealed that mercerization using sodium hydroxide (NaOH) and sodium bicarbonate (NaHCO_3_) improved the mechanical properties of natural-fiber-reinforced composites [14,15,16,17,18]. Moreover, coir-fiber treatment using 5% NaOH aqueous solution for 72 h resulted in a 31% increase in tensile strength of coir–polyester composite [19]. Additionally, treatment of paper sheets formed from recycled paper fibers with NaOH/urea solution improved the physical properties of the sheets [20]. 

### 1.2. Compressive Load

The compression process induces transverse stress that enhances the rearrangement of particles to reduce voids. This process compacts a material by releasing trapped air and reducing the gaps between particles, which improves the material’s properties. Tensile strength can be improved by the application of compressive force in forming pharmaceutical tablets [21,22,23]. Increasing the compaction pressure has also improved the mechanical properties of maize residue pellets [24] and biomass grass pellets [25]. To date, limited literature is available that investigates the effect of compression on the tensile strength of discarded textile and paper waste pulp formed into sheets.

### 1.3. Binding Agents

Binders form strong inter-particle bridges, coatings or films that can improve the strength of a material. Binders can be classified according to the nutritional source, i.e., protein origin, carbohydrate source, and other nutritional contents [26]. Carbohydrate-based binders exhibit a high molecular diversity, which caters for a wide range of applications. These natural biopolymers are potential binders due to their organic properties. 

#### 1.3.1. Molasses

Molasses is a thick dark to light brown syrup with a distinct smell and sweet taste, which is generated as a by-product of sugar cane production. It is widely used as a binder for composite fuel in the form of briquettes or pellets with improved mechanical properties and fuel characteristics [27,28,29]. The recrystallization mechanism of dissolved sugar in molasses at dry state results in an enhanced strength of the pellets [30]. Molasses is typically used as a binder at concentrations ranging from 0% to 20%. Molasses has been used as an additive in papermaking to enhance the strength of paper [31,32]. Furthermore, improvement in physico-mechanical properties of recycled paper made from an old, corrugated container was achieved by using molasses as a dry-strength agent [33].

#### 1.3.2. Sodium Alginate

Alginates are naturally occurring anionic complex polysaccharides derived from the main cell wall of brown seaweeds of Phaeophyceae class [34,35,36]. Alginates are commonly used in many industries because of their unique rheological properties such as thickening, gelling, stabilizing, viscosifying, mucoadhesion, and sol/gel transition ability [37,38]. Among various alginates, sodium alginate is the most common salt of alginate and one of the established biopolymers because of its multifunctional properties and wide range of applications [39,40]. Sodium alginate consists of a complex mixture of oligo-polymers, polymannuronic acid, polyguluronic acid, and a mixed polymer [41]. Moreover, sodium alginate is readily used as a binder for seedling pots because it is biodegradable, biocompatible, widely available, renewable, and non-toxic [42,43]. 

#### 1.3.3. Cornstarch

Starch is a popular biopolymer extensively and widely used as a binder due to its properties, abundance, renewability, and low cost [44,45]. Cornstarch, or corn flour, is the starch extracted from the endosperm portion of the corn or maize kernel. A binder solution can be prepared by dissolving cornstarch in water upon gradual heating to form a paste or gel with increased viscosity. Cornstarch is widely used as a filler or binder in various applications. A cornstarch paste concentration of 5–25% (*w*/*w*) was utilized in tablet granulations [46]. An environmentally sustainable shift from petroleum-based containers to biodegradable containers made of biomass with the incorporation of organic binder like cornstarch is now prevalent. Cornstarch has also been used as a binder in making biodegradable nursery containers [47]. 

## 2. Materials and Methods

### 2.1. Alkali Treatment and Conversion of Waste Materials into Pulp

The substrates tested for this study include textile waste in the form of soiled towel (100% cotton), polycotton fabric (60% cotton and 40% polyester), and paper waste in the form of used newspaper and corrugated cardboard. Analysis of solids, including moisture content, total solids (TS), and volatile solids (VS) content, was conducted for all substrates and binders using standard methods [48]. The substrates were manually cut into small pieces (1 cm × 0.5 cm), weighed, and soaked using NaOH and NaHCO_3_ at 5% and 10% (*w/w*%) for 5 h at room temperature (22 °C). Alkali concentrations at 5% and 10% were considered because the optimum mechanical properties of coir–polyester composite were achieved by using 2–8% *w*/*w* of NaOH [19]. Furthermore, alkali treatment using 10% *w*/*w* of NaHCO_3_ attained the highest tensile strength on fiber-reinforced epoxy composite [14]. 

Thereafter, the substrates were rinsed using deionized water until a neutral pH was achieved, and then subjected to a 2 min pulping process using a 2 L blender (thinkKitchen Pro-vita, 1400 W). The resulting pulp was drained and squeezed to remove excess water using a double folded cheesecloth to avoid pulp wastage and weighed accordingly to form a sheet with 0.5 g TS. The untreated substrate or control was soaked in deionized water for 5 h. 

### 2.2. Preparation of Homogeneous Sheets

The weighed wet pulp from each treatment and substrate type was formed into 5 cm × 2.5 cm × 0.1 cm sheets using a fabricated mold. The mold was drafted using Solid Works software and created by a 3D printer machine using acrylonitrile butadiene styrene (ABS) plastic material. The pulps were placed evenly onto the entire surface of the bottom mold and covered with the top mold. The sheet from each treatment and substrate type was compressed by placing the mold on the platform of a universal testing machine (Model 3366 Universal Testing Systems, Instron Corp., Norwood, MA, USA). Henceforth, a load of 200 N or 500 N was applied to the mold by using a 10 kN load cell at a rate of 10 mm/min. Upon reaching the desired load, the sheet was held inside the mold for one minute to maintain constant pressure and prevent relaxation. Then, the compressed sheet was subjected to drying at 105 °C for 5 h prior to tensile strength testing. 

### 2.3. Binding Agents

Different binders such as blackstrap molasses, sodium alginate, and cornstarch at different concentrations of 5%, 10%, 15%, and 20% on a dry weight basis were blended with the substrate to form six homogeneous sheets. The effect of binders on the tensile strength of homogeneous sheets was tested after employing the optimum results from alkali treatment of 5% NaOH for 5 h of soaking and compression load of 500 N. The binder concentrations of 5–20% were studied as these concentrations were similarly used to determine the effect of molasses on the physico-mechanical properties of old corrugated container recycled paper [33]. Additionally, binders (pitch, molasses, and starch) in the range of 3–20% were studied to determine the densification effect on the composite fuel briquette [29]. Cornstarch as an excipient at concentrations of 5–25% was studied in tablet granulations [46]. Nevertheless, different local binders at concentrations of 5–20% were used to determine the molding properties of silica sand for industrial application [49].

### 2.4. Tensile Strength Test

Tensile strength is an important parameter that indicates the handling capacity of biodegradable seedling containers [50]. In addition, tensile forces are typically exerted on the walls of the container during plant growth and manual transportation [51]. A universal testing machine (LS5 Model, Lloyd Materials Testing, Lloyd Instrument Ltd., West Sussex, UK) equipped with 5 kN load cell was used to determine the tensile strength of the prepared sheets. The top and bottom eccentric roller grips of 50 mm length were attached to the machine that holds the sheet while pulling at a set extension rate (testing speed) of 2 mm/min. The bottom grip was kept fixed while the top grip moves upward during tension. The test was set to stop when the sample breaks to enable break detection. 

## 3. Results and Discussion

### 3.1. Effect of Alkali Treatment

**Figure 1:**
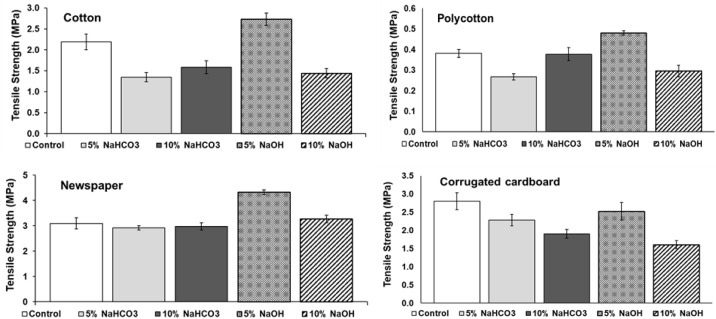
Tensile strength of homogeneous sheets with alkali treatment.

The optimum tensile strength was obtained from 5% NaOH treatment of cotton, polycotton, and newspaper sheets. The highest tensile strength of 4.33 MPa was obtained from newspaper sheets, followed by cotton sheets (2.73 MPa) (Figure 1). The lowest strength of 0.48 MPa was obtained from polycotton. For corrugated cardboard, an optimum tensile strength of 2.80 MPa was obtained from the untreated sheets. Thus, soaking the corrugated cardboard in deionized water is essential for optimal tensile strength.

**Figure 2:**
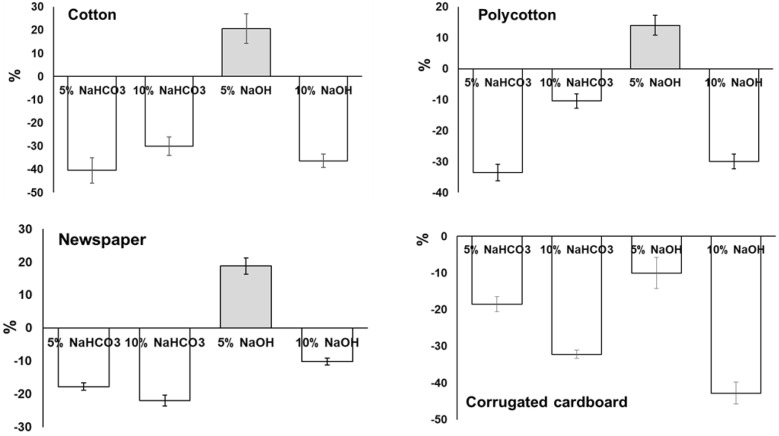
Percent increase or decrease in tensile strength of alkali treated substrates (error bars indicate standard error of the mean).

To quantify the effect of alkali treated sheets vis-à-vis the untreated ones, the optimum results obtained from 5% NaOH were compared to other treatment conditions. It is evident that soaking in 5% NaOH for 5 h increased the tensile strength of the sheets molded from cotton, newspaper, and polycotton by 21%, 19%, and 14%, respectively, compared to the untreated ones (Figure 2). This mild alkali treatment is beneficial to remove impurities from the substrate and can reduce the number of superficial defects, resulting in the improvement of strength [16,17,18], while for corrugated cardboard sheets, alkali treatments were not favorable as there was no improvement in tensile strength. From the results, it can be deduced that 5% NaOH treatment for 5 h of soaking was beneficial for the improvement of tensile strength of cotton, polycotton, and newspaper substrates.

### 3.2. Effect of Compressive Load

**Figure 3:**
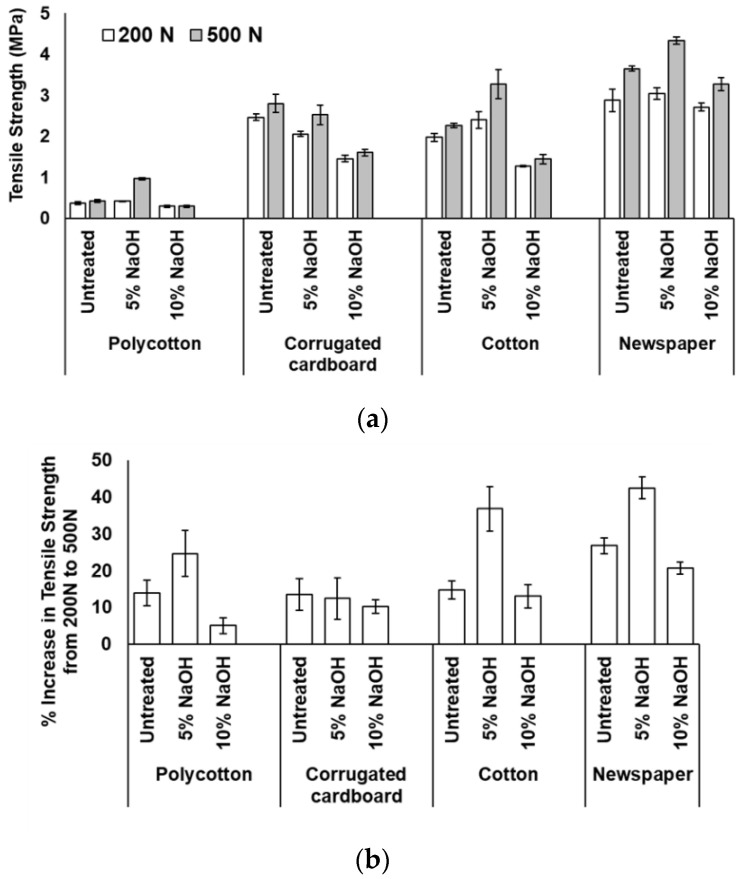
(**a**) Tensile strength and (**b**) percentage increase in tensile strength of compacted sheets at 200 N and 500 N (error bars indicate standard error of the mean).

**Figure 4:**
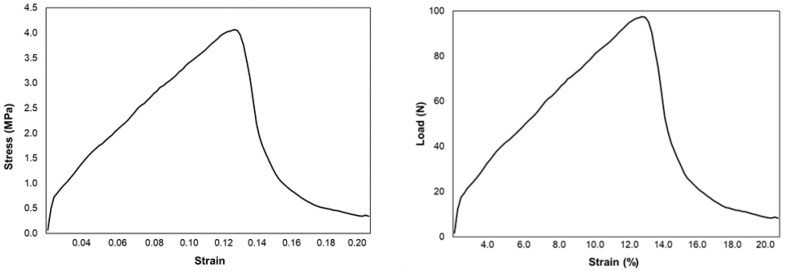
Stress–strain and load–%strain curves of the newspaper sheet.

Figure 3a presents the tensile strength for the four substrates treated with 5% and 10% NaOH (*w*/*w*%), as well as the untreated substrates, under the compressive loads of 200 N and 500 N. The results suggest that for all the substrates tested, the tensile strength improves at a compressive load of 500 N as compared to 200 N. Figure 3b shows the percentage increase in tensile strength from 200 N to 500 N compressive load. The increased compressive load applied to the substrate causes the particles to rearrange, forming a tighter packed structure that minimizes porosity and enhances the tensile strength of the formed sheet [22]. The treatment at 5% NaOH caused the highest increase in tensile strength of 42% in newspaper substrate, followed by cotton and polycotton of 37% and 25%, respectively, while, for corrugated cardboard, the 500 N compressive load was also superior to 200 N, obtaining an optimum tensile strength increase of 12% for untreated sheets. A sample illustration of stress–strain and load–%strain curves for newspaper sheets after alkali treatment and compression at 500 N is given in Figure 4.

### 3.3. Effect of Binding Agents

**Figure 5:**
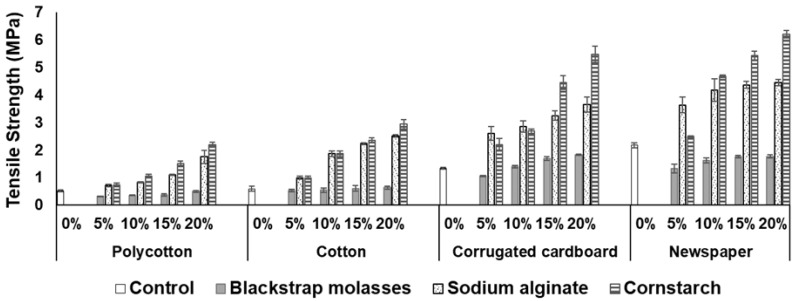
Tensile strength of substrates using different binders (error bars indicate standard error of the mean).

Table 2 presents the solids analyses of the substrates and binders. Cornstarch was the predominant binder in improving the tensile strength of the substrates (Figure 5) among all the binders used. Cornstarch at a concentration of 20% on a dry weight basis per sheet achieved the optimum tensile strength of 2.19 MPa, 2.93 MPa, 5.47 MPa, and 6.20 MPa for polycotton, cotton, corrugated cardboard, and newspaper sheets, respectively. This suggests that the higher tensile strength of newspaper and corrugated cardboard could make it a beneficial material for blending with textiles waste of a lower tensile strength. Moreover, sodium alginate was also effective in improving the tensile strength of the substrates. However, blackstrap molasses as a binder was not effective in improving the tensile strength of the substrates. 

**Figure 6:**
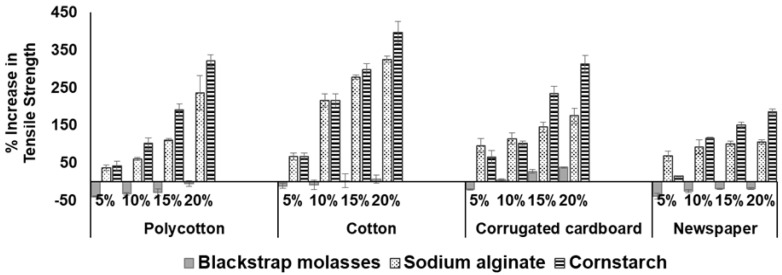
Percentage increase in tensile strength using different binders (error bars indicate standard error of the mean).

To quantify the increase in tensile strength as influenced by the binder, the percentage increase was calculated by using a control (0% binder) as a reference (Figure 6). It is evident that tensile strength increased with increase in binder concentration, particularly for cornstarch and sodium alginate binders. Generally, a direct correlation is typically found between the binder concentration and the tensile strength [52,53,54]. Among the binders, 20% cornstarch provides the highest percentage increase in tensile strength for cotton and polycotton, by 395% and 320%, respectively. Furthermore, in the case of corrugated cardboard and newspaper, the highest increase in tensile strength of 310% and 185% was obtained. Pulp fibers dispersed in a binder matrix enhanced the tensile strength of the resulting material [55].

## 4. Conclusions

This study demonstrates that alkali treatment using 5% NaOH for 5 h increased the tensile strength of cotton, newspaper, and polycotton sheets by 21%, 19%, and 14%, respectively. However, the tensile strength of corrugated cardboard sheets was not enhanced by alkali treatment. Moreover, increasing the compressive load from 200 N to 500 N in forming sheets showed an improved tensile strength of 12%, 25%, 37%, and 42% for corrugated cardboard, polycotton, cotton, and newspaper, respectively. Importantly, the addition of binders demonstrated a significant effect on the tensile strength of the sheets, particularly with the use of 20% cornstarch on a dry weight basis per sheet. The higher tensile strength of newspaper and corrugated cardboard suggests their potential as substrates to be blended with textiles waste of lower tensile strength. The optimum results obtained from this study can expedite future utilization of the waste substrates as biodegradable containers.

## Figures and Tables

**Table 1:** Composition of some fibrous materials.

Fibrous Materials	Cellulose (%)	Hemicellulose (%)	Lignin (%)	Reference(s)
Cotton	82.7	5.7	0.7–1.6	[7,8]
Newspaper	68.5	13.1	23.4	[9]
Corrugated cardboard	52.8	13.3	22.2	[10]

**Table 2:** Solids analyses of waste samples and binders.

Solids Analyses (%)	Substrates				Binders		
	Textile Waste		Paper Waste		Blackstrap Molasses	Sodium Alginate	Corn-Starch
	Cotton	Poly-Cotton	Newspaper	Corrugated Cardboard			
Moisture content	3.93	0.60	3.90	2.60	26.64	10.98	10.03
Total solids	96.07	99.40	96.10	97.40	73.36	89.02	89.97
Volatile solids	99.51	99.52	89.61	91.14	91.89	59.94	99.83
Fixed solids	0.49	0.48	10.39	8.86	8.11	40.06	0.17

## References

[1] USEPA (U.S. Environmental Protection Agency) (2018). Advancing Sustainable Materials Management: 2015 Factsheet. Assessing Trends in Material Generation, Recycling, Composting, Combustion with Energy Recovery and Landfilling in the United States.

[2] Duff S.J.B., Murray W.D. (1996). Bioconversion of forest products industry waste cellulosics to fuel ethanol—A review. Bioresour. Technol..

[3] Chen H., Venditti R.A., Jameel H., Park S. (2012). Enzymatic hydrolysis of recovered office printing paper with low enzyme dosages to produce fermentable sugars. Appl. Biochem. Biotechnol..

[4] Kabir M.M., Wang H., Lau K.T., Cardona F. (2012). Chemical treatments on plant-based natural fibre reinforced polymer composites: An overview. Compos. Part B.

[5] Wallenberger F.T., Weston N. (2004). Natural Fibres, Plastics and Composites.

[6] Rowell R.M., Young R.A., Rowell J.K. (1997). Paper and Composites from Agro-Based Resources.

[7] Fakirov S., Bhattacharyya D. (2007). Engineering Biopolymers; Homopolymers, Blends and Composite.

[8] Mohanty A.K., Misra M., Hinrichsen G. (2000). Biofibres, biodegradable polymers and biocomposites: An overview. Macromol. Mater. Eng..

[9] Yuan X., Cao Y., Li J., Wen B., Zhu W., Wang X., Cui Z. (2012). Effect of pretreatment by a microbial consortium on methane production of waste paper and cardboard. Bioresour. Technol..

[10] Gonzalez-Estrella J., Asato C.M., Jerke A.C., Stone J.J., Gilcrease P.C. (2017). Effect of structural carbohydrates and lignin content on the anaerobic digestion of paper and paper board materials by anaerobic granular sludge. Biotechnol. Bioeng..

[11] Joseph P.V., Joseph K., Thomas S., Pillali C.K.S., Prasad V.S., Groeninckx G., Sarkissova M. (2003). The thermal and crystalline studies of short sisal fibre reinforced polypropylene composites. Compos. Part A Appl. Sci. Manuf..

[12] Li X., Tabil L.G., Panigrahi S. (2007). Chemical treatment of natural fibre for use in natural fibre-reinforced composites: A review. J. Polym. Environ..

[13] Ray D., Sarkar B.K., Rana A.K., Bose N.R. (2001). Effect of alkali treated jute fibres on composite properties. Bull. Mater. Sci..

[14] Fiore V., Scalici T., Valenza A. (2017). Effect of sodium bicarbonate treatment on mechanical properties of flax-reinforced epoxy composite materials. J. Compos. Mater..

[15] Fiore V., Scalici T., Nicoletti F., Vitale G., Prestipino M., Valenza A. (2016). A new eco-friendly chemical treatment of natural fibres: Effect of sodium bicarbonate on properties of sisal fibre and its epoxy composites. Compos. Part B.

[16] Cai M., Takagi H., Nakagaito A.N., Li Y., Waterhouse G.I.N. (2016). Effect of alkali treatment on interfacial bonding in abaca fibre-reinforced composites. Compos. Part A Appl. Sci. Manuf..

[17] Yan L., Chouw N., Yuan X. (2012). Improving the mechanical properties of natural fibre fabric reinforced epoxy composites by alkali treatment. J. Reinf. Plast. Compos..

[18] Goud G., Rao R.N. (2011). Effect of fibre content and alkali treatment on mechanical properties of *Roystonea regia*-reinforced epoxy partially biodegradable composites. Bull. Mater. Sci..

[19] Jayabal S., Sathiyamurthy S., Loganathan K.T., Kalyanasundaran S. (2012). Effect of soaking time and concentration of NaOH solution on mechanical properties of coir-polyester composites. Bull. Mater. Sci..

[20] Miao Y., Chen Y., Jia Q., Bai Z., Shi K. (2018). Water retention and physical properties of recycled fibres treated with NaOH/urea aqueous solution. IOP Conf. Ser. Mater. Sci. Eng..

[21] Kolakovic R., Peltonen L., Laaksonen T., Putkisto K., Laukkanen A., Hirvonen J. (2011). Spray-dried cellulose nanofibres as novel tablet excipient. AAPS PharmSciTech.

[22] Amin M., Albawani S., Amjad M. (2012). A comparative study of the compaction properties of binary and bilayer tablets of direct compression excipients. Trop. J. Pharm. Res..

[23] Juban A., Briancon S., Puel F., Hoc T., Nouguier-Lehon C. (2017). Experimental study of tensile strength of pharmaceutical tablets: Effect of the diluent nature and compression pressure. EPJ Web Conf..

[24] Wongsiriamnuay T., Tippayawong N. (2015). Effect of densification parameters on the properties of maize residue pellets. Biosyst. Eng..

[25] Mani S., Tabil L.G., Sokhansanj S. (2006). Effects of compressive force, particle size and moisture content on mechanical properties of biomass pellets from grasses. Biomass Bioenergy.

[26] De Silva S.S., Anderson T.A. (1995). Fish Nutrition in Aquaculture.

[27] Jittabut P. (2015). Physical and thermal properties of briquette fuels from rice straw and sugarcane leaves by mixing molasses. Energy Procedia.

[28] Zhai Y., Wang T., Zhu Y., Peng C., Wanga B., Li X., Li C., Zeng G. (2018). Production of fuel pellets via hydrothermal carbonization of food waste using molasses as a binder. Waste Manag..

[29] Adeleke A.A., Odusote J.K., Lasode O.A., Ikubanni P.P., Malathi M., Paswan D. (2019). Densification of coal fines and mildly torrefied biomass into composite fuel using different organic binders. Heliyon.

[30] Mišljenović N., Colović R., Vukmirović Ð., Brlek T., Bringas C.S. (2016). The effects of sugar beet molasses on wheat straw pelleting and pellet quality. A comparative study of pelleting by using a single pellet press and a pilot-scale pellet press. Fuel Process. Technol..

[31] Fahmy T.Y.A. (2007). Introducing molasses as a new additive in papermaking. Tappi J..

[32] Fahmy T.Y.A., Mobarak F. (2009). Advanced nano-based manipulations of molasses in the cellulose and paper discipline: Introducing a master cheap environmentally safe retention aid and strength promoter in papermaking. Carbohydr. Polym..

[33] Ashori A., Marashi M., Ghasemian A., Afra E. (2013). Utilization of sugarcane molasses as a dry-strength additive for old corrugated container recycled paper. Compos. Part B.

[34] Pawar S.N., Edgar K.J. (2012). Alginate derivatization: A review of chemistry, properties and applications. Biomaterials.

[35] Holdt S.L., Kraan S. (2011). Bioactive compounds in seaweed: Functional food applications and legislation. J. Appl. Phycol..

[36] Kloareg B., Quatrano R.S. (1988). Structure of the cell walls of marine algae and ecophysiological functions of the matrix polysaccharides. Oceanogr. Mar. Biol. Annu. Rev..

[37] Szekalska M., Puciłowska A., Szymańska E., Ciosek P., Winnicka K. (2016). Alginate: Current use and future perspectives in pharmaceutical and biomedical applications. Int. J. Polym. Sci..

[38] Draget K.I., Moe S.T., Skjak-Bræk G., Smidsrød O., Stephen A.M., Phillips G.O., Williams P.A. (2006). Alginates. Food Polysaccharides and their Applications.

[39] Yoo S., Krochta J.M. (2011). Whey protein–polysaccharide blended edible film formation and barrier, tensile, thermal and transparency properties. J. Sci. Food Agric..

[40] Lee K.Y., Mooney D.J. (2012). Alginate: Properties and biomedical applications. Prog. Polym. Sci..

[41] Smidsrød O., Haug A., Larsen B. (1966). The influence of pH on the rate of hydrolysis of acidic polysaccharides. Acta Chem. Scand..

[42] Avella M., Di Pace E., Immirzi B., Impallomeni G., Malinconico M., Santagata G. (2007). Addition of glycerol plasticizer to seaweeds derived alginates: Influence of microstructure on chemical–physical properties. Carbohydr. Polym..

[43] Immirzi B., Santagata G., Vox G., Schettini E. (2009). Preparation, characterisation and field testing of a biodegradable sodium alginate-based spray mulch. Biosyst. Eng..

[44] Wang Z.J., Li Z.F., Gu Z.B., Hong Y., Cheng L. (2012). Preparation, characterization and properties of starch-based wood adhesives. Carbohydr. Polym..

[45] Tabasum S., Younas M., Zaeem M.A., Majeed I., Majeed M., Noreen A., Iqbal M.N., Zia K.M. (2019). A review on blending of corn starch with natural and synthetic polymers, and inorganic nanoparticles with mathematical modeling. Int. J. Biol. Macromol..

[46] Rowe R.C., Sheskey P.J., Owen S.C. (2006). Handbook of Pharmaceutical Excipients.

[47] Sun E., Huang H., Sun F., Wu G., Chang Z. (2017). Degradable nursery containers made of rice husk and cornstarch composites. BioRes.

[48] Rice E.W., Bridgewater L. (2012). Standard Methods for the Examination of Water and Wastewater.

[49] Joshua T.O., Fayomi O.S.I., Olatuja F.H. (2016). Hybrid effect of selected local binders on the moulding properties of River Niger silica sand for industrial application. J. Nanosci. Adv. Technol..

[50] Castronuovo P., Picuno D., Manera C., Scopa A., Sofo A., Candido V. (2015). Biodegradable pots for pointsettia cultivation: Agronomic and technical traits. Sci. Hortic..

[51] Stone P. (2017). Evaluation of Biosolids for Use in Biodegradable Transplant Containers. Master’s Thesis.

[52] Alebiowu G., Itiola O.A. (2003). The effects of starches on mechanical properties of paracetamol tablet formulations. Acta Pharm..

[53] Chen G., Zhu Z.J., Salminen P., Toivakka M. (2014). Structure and mechanical properties of starch/styrene-butadiene latex composites. Adv. Mater. Res..

[54] Okoye E.I., Onyekweli A.O., Ohwoavworhua F.O., Kunle O.O. (2009). Comparative study of some mechanical and release properties of paracetamol tablets formulated with cashew tree gum, povidone and gelatin as binders. Afr. J. Biotechnol..

[55] Curvelo A.A.S., de Carvalno A.J.F. (2001). Thermoplastic starch-cellulosic fibers composites: Preliminary results. Carbohydr. Polym..

